# Sharing Is Caring: Helping Institutions and Health Organizations Leverage Data for Educational Improvement

**DOI:** 10.5334/pme.1081

**Published:** 2024-10-07

**Authors:** Stefanie S. Sebok-Syer, Alina Smirnova, Ethan Duwell, Brian C. George, Marc M. Triola, Christopher A. Feddock, Saad Chahine, Jonathan D. Rubright, Brent Thoma

**Affiliations:** 1 Stanford University, United States; 2 Department of Family Medicine, University of Calgary, Canada; 3 Biomedical Engineering, Medical College of Wisconsin, United States; 4 University of Michigan, United States; 5 NYU Grossman School of Medicine, United States; 6 Educational Strategy, National Board of Medical Examiners, United States; 7 Faculty of Education, Queen’s University, Canada; 8 Office of Research Strategy, National Board of Medical Examiners, United States; 9 University of Saskatchewan, Canada; 10 School of Medicine, Toronto Metropolitan University, Canada

## Abstract

Competency-based medical education (CBME) has produced large collections of data, which can provide valuable information about trainees and medical education systems. Many organizations continue to struggle with accessing, collecting, governing, analyzing, and visualizing their clinical and/or educational data. This hinders data sharing efforts within and across organizations, which are foundational in supporting system-wide improvements. Challenges to data sharing within medical education include variability in legislation, existing data policies, heterogeneity of data, inadequate data infrastructure, and various intended purposes or uses. In this eye opener, the authors describe four case studies to illustrate some of the aforementioned challenges and characterize the complexity of data sharing within medical education along two dimensions: organizational (single vs. multiple) and data type (clinical and/or educational). With the goal of better supporting data sharing initiatives, the authors introduce an action-oriented blueprint that includes a three-stage process (i.e., preparation, execution, and iteration) to highlight crucial aspects of data sharing. This evidence-informed model incorporates current best practices and aims to support data sharing initiatives within their own organizations and across multiple organizations. Finally, organizations can use this model to conceptually guide and track their progression throughout the data sharing process.

## Introduction

The shift to competency-based medical education (CBME) has produced large amounts of data that reflect trainees’ performance. Harvesting these data can provide information and insights not only about trainees, but also about our systems as a whole [1]. At the organizational level, analyses of educational and clinical data can inform trainees’ teaching and learning [2,3], support promotion decisions [4], guide faculty development [5], and enhance program evaluation and quality improvement [6]. At the system level, analyses of aggregated data can guide the development and design of assessment programs [1], quantify the impact of education on patient outcomes [7,8], inform credentialing of programs and individuals [9], and shape accreditation standards [1]. Despite the promise and potential of these data, many organizations continue to grapple with challenges accessing, collecting, amalgamating, governing, analyzing, and visualizing their data for educational purposes. Furthermore, these struggles constrain data sharing efforts across organizations, which are ultimately needed to support system-wide improvements.

Numerous challenges exist that prevent data sharing efforts within medical education. First, governance and oversight of educational and clinical data are generally guided by legislation that varies between jurisdictions, thereby complicating data sharing efforts. Second, most training programs function in conjunction with a hospital system or higher education organization, which tends to result in the adoption of existing clinical and/or educational data policies, protocols, governance, and information systems with little to no consideration of how medical education data needs fundamentally differ. Finally, the heterogeneity of data across organizations, differences in data infrastructure, and various intended uses for these data make it next to impossible to develop universal solutions to these problems. Some organizations have invested significant resources to retrofit solutions, while others remain stalled as to how to address these challenges, thereby limiting data sharing efforts within and across organizations.

The root cause of many challenges in medical education is the general callowness in collecting, analyzing, and visualizing large amounts of data. In this eye opener, we leverage our team’s interdisciplinary expertise and draw upon their first-hand experiences to introduce an action-oriented blueprint that can be used to guide and track data sharing efforts within medical education. Our goal is to bridge the gap between medical educators and technological support staff so that each organization can develop and implement an effective data sharing strategy that affords them the opportunity to share data within and across organizations.

### Definitions and Terminology

Articulating the complexities of data sharing within medical education is particularly challenging because of variations in terminology and the fact that terms are often used interchangeably. For clarity and consistency, we begin by presenting a glossary of terms used throughout this piece (Appendix A). We use the term *organization* to broadly encompass those parties involved in the provision of medical education (e.g., educational programs and institutions, health systems, and regulatory bodies). We define *data sharing* as the exchange of data between any organizations and utilize the term *data amalgamation* to refer to the integration of data from multiple sources within or across an organization.

## Case Studies Illustrating the Complexity of Data Sharing

**Figure 1:**
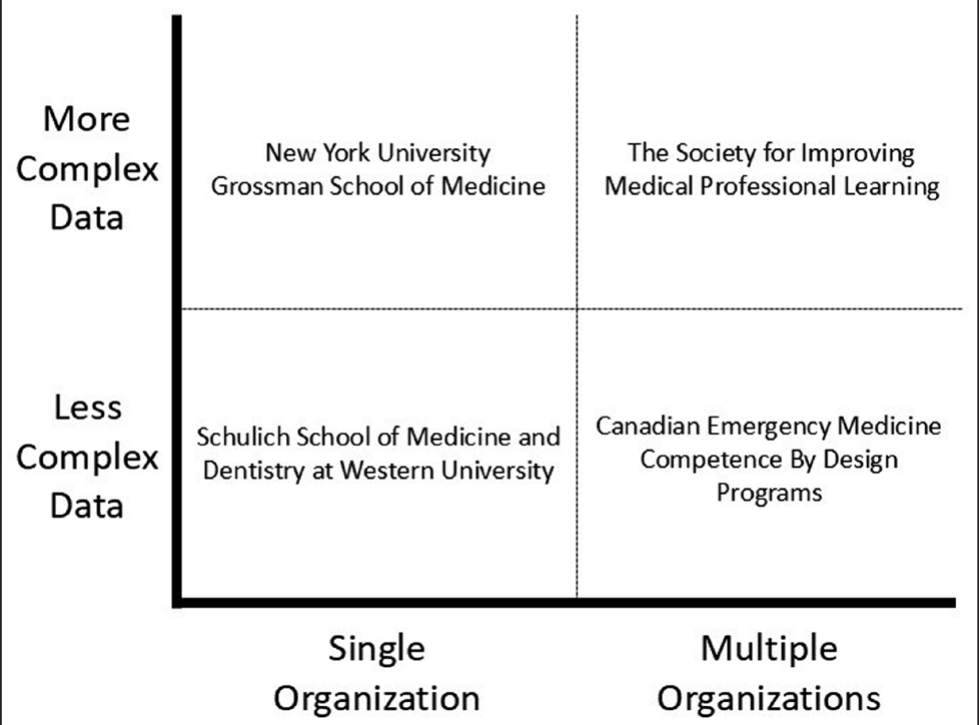
Diagram depicting the organizational complexity and data complexity of four different case studies.

Using four different case studies, we illustrate the breadth of data sharing possibilities within medical education. These cases were deliberately selected to showcase real-world examples of data sharing and highlight challenges that these organizations faced throughout the process. Figure 1 visualizes the complexity of data within medical education along two dimensions. On the horizontal axis, we have organizational complexity, categorized as data originating from a single organization (left) or multiple organizations (right). While on the vertical axis, we have data complexity that describes the source(s) of data. For example, data can be clinical (e.g., electronic health record) or educational (e.g., assessments based on direct observations) and can come from a single source (bottom) or be more complex, incorporating some combination of clinical and educational sources (top). The quadrants identified using these two dimensions are valuable because they help an organization assess their capacity for data sharing. It is also worth noting an additional challenge (not depicted): we currently lack a robust data system that allows for seamless integration of trainees’ data as they move across educational and clinical organizations over time.

Our first case study – *Schulich School of Medicine and Dentistry at Western University* – describes one of the simplest use cases of data sharing within medical education: one source of data from a single organization [10]. The Division of Emergency Medicine (EM) within the Schulich School of Medicine and Dentistry at Western University in Canada represents one of the earliest examples of using clinical data to support educational assessment. Starting in 2015, all attendings in the Division of EM routinely received clinical report cards containing electronic health record (EHR) data metrics that depicted their individual performance. In 2017, the educational team at Western University leveraged their existing relationship with the hospital information technology (IT) unit to expand their clinical data sharing to include residents. Some of the resident EHR metrics were the same as the faculty, but some were distinct, reflecting the unique role of residents in our healthcare system. Using action research methodology [11], education scientists elicited the opinions of faculty and residents to adapt existing clinical measures and develop new resident-specific clinical measures. For example, one metric – *time to first lab order* – was created because residents felt it was meaningful to capture clinical care moments where they had the greatest opportunity to perform individually [12]. Throughout this process, the expertise of educators (i.e., education scientists and clinician educators) was vital in ensuring that the clinical data extracted from the EHR databases were accurate and meaningful in supporting the educational mission and commitment to teaching and assessment. While this effort did not require the data sharing outside the hospital system, the support of organizational leaders was essential to moving the project forward and ensuring the use of the clinical report card within the training program. By securing the support of clinical and educational leaders first, our team was able to develop and implement the resident clinical report card iteratively and in parallel. Finally, given that clinical data were used for educational purposes within a single organization, no formal data sharing agreements were required.

Our second case study – *New York University Grossman School of Medicine* – depicts an example of data amalgamation (i.e., from multiple sources of data) and sharing within a single organization [13]. New York University (NYU) Grossman School of Medicine is one of the first documented organizations to successfully collect, amalgamate, and store both educational and clinical data so that it can be used for a variety of purposes, thereby supporting the clinical and educational mission of NYU. Pioneered by the Institute for Innovations in Medical Education, its development was championed by an MD clinical informatics medical educator, the *Education Data Warehouse* represents an integrated system and centralized repository of all data pertaining to medical education across the continuum of medical students, residents, and faculty. Collating clinical data from the EHR (i.e., EPIC’s chronicles, clarity, caboodle) with trainee outcomes, assessment data from faculty, and the content of the curriculum enables combined analyses and visualizations of these data using sophisticated dashboards. More recent applications of these data include facilitating the transition from undergraduate to graduate medical education and supporting faculty and trainee coaching [14,15]. By actively engaging various stakeholders and leveraging the knowledge and experience of administrators, educators, and IT personnel at NYU, these trainee and leadership dashboards serve both strategic, instructional, and evaluative purposes. NYU has established partnerships with several hospitals and residency programs within their organization. For example, NYU has developed shared data access from NYU Langone Health, NYU Long Island, and NYU Brooklyn, which are separate residency programs each with their own set of hospitals, for the purposes of examining larger clinical and educational patterns in the New York City area. A key factor in making this possible is mission alignment, which is simplified at NYU given that the Dean of NYU Grossman School of Medicine is also the CEO of NYU Langone Health. Furthermore, the early development of the Education Data Warehouse was performed by faculty and staff with a background and experience in clinical informatics and aligned with the mission of NYU Grossman School of Medicine. As data demands of various stakeholders increase, the need for data governance becomes essential in ensuring that high standards exist for data stewardship, quality, and management, while also continuing to align with the mission “to serve, to teach, and to discover.”

Our third case study – *Canadian EM Competence by Design (CBD) Programs* – showcases educational data sharing between numerous organizations [16]. The Royal College of Physician and Surgeons of Canada’s (RCPSC) CBD project created a consistent, specialty-specific programmatic assessment guide that was implemented nationally. To evaluate implementation of this assessment program across sites and inform its evolution, a research group including program directors from most EM programs throughout the country committed to sharing assessment data from the 2018 cohort of residents. Despite agreement regarding the importance of the initiative as well as the benefits of developing a unified, consistent assessment plan, mobilizing data sharing efforts between organizations was challenging. The main issue being that the data architecture differed from organization to organization thereby limiting interoperability. Some sites also had difficulty extracting the data from their own learning management systems or concerns related to sharing resident-level data. Ultimately, these complications resulted in only quantitative, aggregate, program-level assessment data being shared. As only aggregate analytics were shared, raw data was not transferred and data sharing agreements between the sites were not required. Although this limited resident-level analytics and prevented analysis of narrative assessments, it preserved the anonymity of residents’ assessments and allowed 15 of 17 Canadian EM programs who participated feel comfortable. Getting to a place where even some exchange of data occurs can be a precursor to more ambitious data sharing efforts in the future. Unfortunately, plans for subsequent data collection and analyses were disrupted by the COVID-19 pandemic and the development of a multi-specialty, multi-site pilot focused on the sharing of deidentified disaggregated assessment data that was informed by this early work.

Our fourth case study – *The Society for Improving Medical Professional Learning* – describes a partnership between multiple organizations, which enables sharing of both clinical and educational data [17]. The Society for Improving Medical Professional Learning (i.e., SIMPL) is an international network of training programs that work collaboratively to implement an evidence-based educational system. This SIMPL collaborative was established in an effort to overcome the barriers of implementing a competency-based medical education system [18]. Groups of medical educators within Surgery voluntarily agreed to share their collective resources, which includes their data, to contribute to the development of an infrastructure that supports the collection and sharing of multi-stakeholder data. By contributing to this effort, members of the SIMPL collaborative have access to all the data that exist within the SIMPL network, which helped create interest across multiple organizations. Their work is financed primarily by contributions from organizational members based on a shared vision and interests (i.e., crowdsourcing). Data collection and, by virtue of design, data structure is standardized with all members using a common measurement platform; however, there is still variation in how the platform is used across organizations. In addition to using this data for educational quality improvement purposes, individual trainees and faculty can also consent to have their data used for research. To join the consortium, educational programs must also consent to share their deidentified data with other members for research and improvement purposes. SIMPL data are governed by committees of medical educators and guided by explicitly defined principles for data sharing. Over time, multiple protections for individuals and programs were developed, mostly in response to member feedback. SIMPL received a designation from the Agency for Healthcare Research and Quality as a patient safety organization, which protects sensitive provider and patient data from being used in malpractice litigation. These strategies enabled SIMPL to collect and share increasingly complex data from multiple organizations to support large-scale research and improvement initiatives. Recently, SIMPL expanded and is a hub for data collection and sharing of assessments within and across other specialties (e.g., EM) [19].

## The Eye Opener: Our conceptual model for sharing educational and clinical data

**Figure 2:**
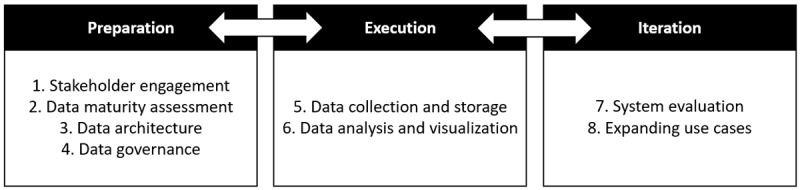
Blueprint of the steps to enable data sharing within medical education.

We developed an action-oriented blueprint to organize critical steps required when sharing clinical and educational data within medical education. This blueprint outlines our conceptual model for data sharing (Figure 2), which is helpful for bridging the gap between educators, clinicians, and IT experts. Organizations can use this three-stage process (Preparation, Execution, and Iteration) to conceptually plan and frame their progression throughout the data sharing process. Those organizations that have yet to amalgamate their own data may also find this model useful given the broad applicability of the principles. To increase the likelihood of success, we recommend starting with internal data amalgamation and small-scale data sharing before initiating higher complexity data sharing. Particular recommendations for navigating organizational complexities will vary based upon the circumstances and are beyond the scope of this manuscript; however, guidelines for the development of any multi-institutional leadership challenges are applicable.

In this section, three stages of the data sharing process are described. Before initiating any data sharing project, an organization needs to consider what expertise and skills are required in order for the project to succeed. For example, with the Schulich School of Medicine and Dentistry at Western University case study, the ability to leverage the expertise of the engineers in the hospital IT unit was crucial to successfully implementing data sharing as the EM residency did not have this expertise in their program. Specific domain expertise and skillsets for data sharing are described in Table 1 and referred to throughout our explanation of the conceptual model.

**Table 1:** Domain expertise and skillset required to support data sharing within medical education.


Domain Expertise	Skillset	Rationale

Mathematics and Statistics	Expertise in advanced data analysis and presentation.	The analysis and visualization of data are required to develop analytics and models that translate data into information.

Computer Science and Information Technology (IT)	Expertise in computer programming and data science.	The amalgamation, organization, storage, maintenance, visualization, and transfer of data require specialized knowledge to develop and implement sustainable data infrastructure.

Medical Education	Expertise in curriculum, teaching and learning, assessment, program evaluation, policy, and quality improvement.	The ability to effectively use and share data requires an understanding of where data comes from and how data are produced and represented in a given context.

Operations	Expertise in project management, design, accounting, and law.	The individuals responsible for supporting the operational aspects of data sharing help ensure the project is completed on time, within budget, and adheres to compliance with pertinent regulations.


### Step 1: Preparation

The preparation stage includes identifying and engaging stakeholders, determining the maturity of data and IT systems, designing a data architecture, and developing a data governance framework. In this section, each step is identified, and the various aspects within each step are explained in greater detail.

#### Stakeholder Engagement

When working with data it is important that all relevant stakeholders are identified and engaged early in the process. Engagement of stakeholders not only lends support but allows for meaningful contributions of diverse perspectives. Some stakeholders to consider include: 1) individuals in positions of authority, usually with ownership over data, 2) individuals responsible for oversight and accountability (e.g., ethics review boards or those responsible for data sharing agreements and governance), and 3) individual users of shared data. While stakeholder analysis has morphed into its own discipline [20], within medical education organizational stakeholders are broadly divided into three groups:

- **Organizational Leaders:** These stakeholders oversee data, resources, and policy. They are generally leaders within an organization that have implicit or explicit oversight over the data and resources required for a data sharing initiative to move forth. They sometimes initiate the work, often have a vested interest, and may even be end-users. Their support and endorsement are essential for all data sharing initiatives.
- **IT Personnel:** These stakeholders are responsible for managing an organization’s software and hardware resources. They are distinct from members of the implementation team as they have roles that will impact an organization’s ability to share data but are not always formal team members.
- **End-users:** These stakeholders use the data and are the perceived beneficiaries of a data sharing initiative. Within medical education, the end-users may include trainees, faculty, competence committees, faculty developers, quality improvement experts, accreditation bodies, and credentialing bodies. Understanding what each of these end-users need from the data to support their work is imperative.

One challenging aspect of stakeholder engagement is what to do with differing opinions. To mitigate these issues, we suggest meeting stakeholders where they are at, listening and trying to understand why a particular stakeholder group holds their perspective, and looking for places where different perspectives align. Areas of mutual interest can be a great starting point for fostering communication and collaboration. The NYU Grossman School of Medicine case study nicely illustrates how to capitalize on those instances of stakeholder alignment to move data sharing initiatives forward.

#### Data maturity assessment

A data maturity assessment [21,22] should be performed to understand the technical capabilities that currently exist. This should be done before implementing any data sharing initiative because not having the appropriate infrastructure will cause a bottleneck. Domain expertise in computer science and IT will help define how data are currently being collected and/or used, which provides insights into what is available as well as what will be needed in the future. In some situations, organizations will discover that they do not have the infrastructure needed to meet the needs of an initiative. In these instances, it is important to explore all possible options as simply creating a data infrastructure can be cumbersome and expensive. The SIMPL case study illustrates one possible solution to this problem, which is to collaborate and share resources with those who do have an established data infrastructure. This approach also allows organizations time to develop a plan to mature their data domains. As part of this development approach, breaking data aspects out into distinct categories (e.g., at risk, foundational, intermediate, and advanced) helps to determine what aspects need to be prioritized. Finally, while expertise from within organizations may provide useful insights, there are nuances surrounding the complexity of data sharing in medical education that differentiate it from purely educational or clinical data. Domain expertise in medical education can also be helpful to liaise between various stakeholders and a project team in a way that ensures all stakeholder voices are heard.

#### Data Architecture

Along with data maturity, domain expertise in computer science and IT is required to develop a data architecture that translates the needs of an organization into tangible requirements for the data system. Data architecture describes how data are acquired, transported, stored, queried, and secured, which is important to consider early because decisions made early can have implications later. Data architecture refers to data models, processing, and interoperability and considers where data are generated (e.g. the software that collects educational data), how data are warehoused and organized (including the data specifications describing how data are formatted), tools that will analyze data, and connections between each of these components. A detailed description of one’s data architecture will describe the scope and cost of the project in addition to providing details about how data sharing will work. Operational expertise can be leveraged to determine the initial and ongoing costs for a proposed solution and any proposed alternatives. For example, a contrast can be made between the NYU case (where a sophisticated data architecture was established) and the Canadian EM CBD programs (which relied upon the amalgamation of aggregate data within individual sites, limiting both the need for a sophisticated data architecture as well as the ability to perform more complex analyses).

#### Data Governance

In addition to data architecture, a data governance framework should be developed to a) classify the data and its sensitivity, b) outline how data will be protected, and c) describe how quality assurance will be maintained. As referenced by NYU Grossman School of Medicine and SIMPL case studies, data governance is vital to scale data sharing across organizations and drive policy. Data governance can be considered in terms of the principles upheld and evaluated along five dimensions [23]:

1. **Stakeholders:** those affected by or who have an influence on the way data is governed.
2. **Governance goals:** the stated objectives of stakeholders.
3. **Value from the data:** recognition of the value that any data generates and how such value is distributed to stakeholders.
4. **Governance mechanisms:** the instruments used to achieve governance goals.
5. **Reciprocity:** the relationship between stakeholders for data access and use.

In several instances, data governance structures are defined through legislation and many organizations have pre-existing documentation that one can use to outline a data governance framework. Although data governance should be created and communicated in the early phases of data sharing, it is also important to review data governance structures for specific functions and stakeholder alignment throughout any data sharing initiative. Cornell University, for example, has a comprehensive example of a data governance framework that highlights data sharing across different collaborative organizations [24].

### Step 2: Execution

The execution phase involves initial collection, amalgamation, and storage of data as well as analysis and visualization. What is important to understand with this step is that despite well-formulated plans this stage usually requires several iterations to effectively meet the needs of end-users.

#### Data collection, amalgamation, storage, and sharing

Collection, amalgamation, storage, and sharing is challenging regardless of whether data come from various internal systems or are amalgamated from outside systems. Amalgamated data needs to be reorganized and/or tagged before it can be added to a unified database. Domain expertise in computer science and IT is instrumental throughout this process because tests of both the assumptions made about data in the preparation stage and alignment of datasets with data standards are connected. Depending upon the messiness and variability of data, cleaning and refining can be resource intensive. When amalgamating and sharing data, investment in the automation of repetitive tasks using artificial intelligence (AI) tools can improve the sustainability of the initiative. Finally, when engaging in multi-organizational data sharing, determining who is responsible for completing different aspects of this work should be discussed, negotiated, and solidified as early as possible.

#### Data analysis and visualization

Ideally, analysis and visualization of data are customized for each use case. There are several approaches to this challenge, which frequently require domain expertise from both mathematics and statistics as well as computer science and IT. The custom design of analytics and visualizations is most versatile; however, these vary upon the skillset of the programmer and are more expensive to develop and support. Conversely, many organizations have access to one or more packaged data analytics and visualization tools (e.g. PowerBI, Tableau), which are not as flexible, but rely less on domain expertise and skillsets. Despite these tools not being nearly as versatile, they can often meet the needs of most users and are less expensive to build and maintain. Most notably, those with domain expertise in medical education can often be leveraged to ensure data are reflected and visualized in a meaningful way.

### Step 3: Iteration

Through the process of data sharing, stakeholders begin to realize the potential data have on various aspects of their work. Organizational leaders should plan and secure support for these initiatives early to ensure that any local or external collaborators involved in the initial development of the system can be sustained and participate in ongoing improvements. In this step, operational domain expertise from leadership is required to prioritize various stakeholder requests.

#### Systems Evaluation and Quality Improvement

Iterations related to the expansion of analytics and visualizations are necessary to address evolving stakeholder needs. The initial evaluation of the system should first ensure that data initiatives are meeting the needs of current stakeholders and those who prompted development. Viewing analytics and visualizations will elucidate new ideas for how data can be organized and interpreted. Individuals should anticipate spending a significant amount of time evaluating and improving the analytics and visualizations to meet stakeholders’ needs. For example, requests for visuals supporting quality improvement of educational and/or clinical programming by presenting year-over-year performance may not be needed initially but are likely to be requested as soon as multiple years of data are collected. Using a crawl, walk, run approach [25] can help organize these aspects as data sharing initiatives expand and evolve in ways that plan for iterations [21,22] that shift the focus from descriptive to diagnostic and predictive [26].

#### Expanding Use Cases

Requests for new analytics and visualizations will likely result from integration of new or expanded needs. These iterations will be more difficult to manage because they are difficult to anticipate within the design of a data architecture. As an example, one of the authors (BT) developed a dashboard to support competence committee promotion decisions [4] and before this was completed, he received additional requests to develop dashboards that optimize data for resident learning [2], faculty development [5], and program evaluation [6]. The triage and prioritization of these requests is particularly challenging. In this case, requests for new features were collected formally through stakeholder interviews and focus groups before being thematically analyzed to understand both the request and the underlying needs of the individual(s) making the request. This analysis helped to determine the urgency and impact of the requested change and, along with an analysis of the resources required to add the feature, the leadership team was able to decide on if and when it would be prioritized. While most organizations will not require such a formal process, developing a workflow that collects requests, understands the underlying reason for the request, and considers resource implications is key to ensure effective triage and prioritization of scarce resources. What can be even more challenging is that these expanded capabilities require further amalgamation and often sharing of data from different sources. For example, the integration of clinical and educational data could be incredibly synergistic, but as illustrated with our case studies can become substantially more complex (Figure 1). The integration of milestones assessment data from a trainee’s residency program with performance metrics from that trainee’s clinical practice is twofold: it can provide a more complete depiction of the trainee’s performance and offer insight into the validity of the milestone metrics. From a data standpoint, this requires linking educational data with some clinical database. Although worthwhile, such linking can be incredibly challenging – particularly if trainees provide clinical care in multiple organizations. Throughout the iterative process, one must remain vigilant and revisit elements from the preparation and execution stage to ensure that these can be updated to reflect ongoing alignment with any changes.

Finally, as the field of medical education continues to expand our data sharing capabilities our ability to address ethical and regulatory issues becomes increasingly important [27,28]. The rise of data-hungry AI models over the past year has raised concerns regarding the appropriate use of educational and clinical data [27]. Recent manuscripts have highlighted best practices for data sharing in health research [29] and health professions education [30] that provide up-to-date guidance, much of which is focused on ensuring that when trainee and patient data are shared safeguards exist for these vulnerable groups. Kulasegaram and colleagues’ [30] consensus statement is notable for its advocacy for a) governance bodies providing oversight and accountability, b) transparency and informed consent, and c) respect for autonomy and equity, which are all aimed at addressing ethical issues when sharing data.

## Conclusion

While we initially conceived this piece as a way of proposing guidelines to facilitate data sharing in medical education, no clear, uncomplicated approach applies in all situations. Data sharing is not a field of absolutes, but rather a field of ‘sort-ofs’, ‘maybes’, and ‘it depends’. Furthermore, we found limited research from related fields that could be readily translated to our medical education context. While aspects of clinical care and medical education have parallels to other fields and industry, only medical education combines performance data and educational workplace-based assessment data in multi-year, multi-organizational training environments. As a result, this evidence-informed guide for data sharing incorporates best practices to support those attempting data sharing initiatives within their own organizations.

## Appendix A. Glossary of Terms

**Table:**

Data architecture	A data architecture contains the models, policies, and standards for how data is collected, stored, arranged, and used within an organization.

Data aggregation	Data aggregation is the reporting or presentation of data at a summary level to protect individuals, programs, or organizations

Data amalgamation	Data amalgamation is the integration of data from multiple sources. Data will frequently need to be amalgamated both within a single organization before it is shared and when it is integrated with data shared by other organizations.

Data governance	Data governance outlines the processes, roles, responsibilities, and standards that ensure the quality and security of an organization’s data.

Data maturity assessment	An analysis of an organization’s current data processes and their ability to meet their needs.

Data sharing	Data sharing is the exchange of data between organizations or the transfer of data from one organization to another.

Educational data	Data collected within the course of medical education activities that has implications for teaching and learning

Interoperability	In reference to data, interoperability is the ability to share and amalgamate data from more than one source seamlessly due to clear and consistent standards for the contents, context, and meaning of data.

Stakeholder	We consider a stakeholder to be any individual that contributes, manages, or uses data within an organization.
